# Patient-Reported Flare Symptoms Following COVID-19 Vaccination in Systemic Lupus Erythematosus

**DOI:** 10.7759/cureus.110457

**Published:** 2026-06-08

**Authors:** Pannipa Bupparenoo, Kittiwan Sumethkul, Sungchai Aungthararak

**Affiliations:** 1 Rheumatology and Allergy Unit, Department of Internal Medicine, Rajavithi Hospital, College of Medicine, Rangsit University, Bangkok, THA

**Keywords:** autoimmune disease, covid-19 vaccines, disease flares, systemic lupus erythematosus, vaccination safety

## Abstract

Introduction: Disease flare after COVID-19 vaccination is a major concern among patients with systemic lupus erythematosus (SLE) and may contribute to vaccine hesitancy. We evaluated patient-reported flare symptoms, associated factors, and adverse events following COVID-19 vaccination in patients with SLE.

Methods: We conducted a cross-sectional study of adult patients with SLE who received at least one COVID-19 vaccine dose. Demographic data, disease activity, treatments, and patient-reported flare symptoms occurring within four weeks after vaccination were collected.

Results: A total of 324 patients were included; 94.4% were women with a mean age of 40.8 ± 11.7 years. Most patients received at least two vaccine doses (92.0%), and 66.0% received three doses. Patient-reported flare symptoms occurred in 17.0% of patients. The most common manifestations were joint pain, fatigue, hair loss, and myalgia. Most patient-reported flares were mild, though 9% required hospitalization. Current prednisolone use was associated with patient-reported flare symptoms (p = 0.049), though this borderline association should be interpreted cautiously. Vaccine type and baseline disease activity were not associated with patient-reported flare.

Conclusions: Patient-reported flare symptoms following COVID-19 vaccination were generally mild in this SLE cohort. Vaccine type was not associated with patient-reported flare symptoms, supporting the overall tolerability of repeated COVID-19 vaccination in patients with SLE.

## Introduction

The COVID-19 pandemic has posed a substantial global health burden. Patients with systemic autoimmune rheumatic diseases, including systemic lupus erythematosus (SLE), may experience more severe outcomes due to immune dysregulation and the use of immunosuppressive therapies [1-4]. Vaccination has, therefore, been strongly recommended as the primary preventive strategy [5-9]. Current guidance from the Centers for Disease Control and Prevention continues to recommend COVID-19 vaccination, including booster doses, for immunocompromised individuals such as patients with SLE [10].

Despite these recommendations, vaccine hesitancy remains a concern, particularly due to fears of disease flares following vaccination [11]. These may discourage patients from receiving vaccination [12,13]. Several studies have evaluated post-vaccination disease flares in patients with SLE; however, most were conducted in Western populations predominantly receiving mRNA vaccines and often relied on physician-confirmed outcomes [13-17].

Data from Southeast Asia remain limited, particularly regarding patient-reported experiences and heterogeneous vaccine platforms including inactivated vaccines, which were widely used during the pandemic. Therefore, this study aimed to evaluate patient-reported disease flare symptoms and adverse events following COVID-19 vaccination in patients with SLE in a real-world setting in Bangkok, Thailand.

## Materials and methods

Study design

This cross-sectional study was conducted at a tertiary referral center (Rajavithi Hospital, Bangkok, Thailand) between January and October 2022. Medical records of patients with SLE who received COVID-19 vaccination were reviewed.

Patients

Patients aged ≥18 years with a diagnosis of SLE according to the 1997 American College of Rheumatology (ACR) revised criteria [18], the 2012 Systemic Lupus Erythematosus International Collaborating Clinics (SLICC) criteria or the /2019 ACR/European Alliance of Associations for Rheumatology (EULAR) criteria for SLE were eligible [19,20]. Patients who had received at least one dose of a COVID-19 vaccine during the study period were included. Patients with SLE overlapping with other autoimmune rheumatic diseases (except for antiphospholipid syndrome and Sjögren’s syndrome), inability to complete the interview, pregnancy, and receipt of any other vaccines within 1 month following COVID-19 vaccination were excluded.

Main outcome variable

The primary outcome was patient-reported flare symptoms following COVID-19 vaccination. Patient-reported flare symptoms were defined as new or worsening SLE manifestations [21] occurring within four weeks after vaccination, based on patient-reported symptoms obtained through structured interviews. Physician confirmation or systematic laboratory reassessment was not routinely available; therefore, outcomes represent patient-reported disease worsening rather than confirmed clinical flares.

Study factors

Potential predictive factors for disease flare included demographic characteristics, baseline disease activity status, and immunosuppressive medications at the time of vaccination. Disease activity prior to vaccination was assessed using the Clinical Systemic Lupus Erythematosus Disease Activity Index 2000 (cSLEDAI-2K) [22].

Disease activity was retrospectively evaluated within six months before vaccination and categorized into three groups: Clinical Remission (CR), defined according to the Definitions of Remission in SLE (DORIS) [23], as a cSLEDAI-2K score of 0 (excluding complement and anti-dsDNA domains) with stable maintenance therapy, including antimalarials with or without prednisone ≤5 mg/day, immunosuppressives, and/or biologics; Low Disease Activity (LDA), defined as a cSLEDAI-2K ≤4 with prednisolone ≤7.5 mg/day or equivalent and maintenance antimalarials and/or immunosuppressive agents [24]; and Not in CR/LDA.

Other variables

Other variables collected included age, sex, disease duration, comorbidities, vaccination type, number of vaccine doses received, and concomitant medications such as glucocorticoids, conventional synthetic disease-modifying antirheumatic drugs, and biologic agents.

Procedures

Patient-reported flare occurrence was assessed using patient-reported questionnaires. Clinical, laboratory data, and baseline disease activity were obtained from electronic medical records; therefore, the number of patients tested varied across laboratory parameters.

Ethical considerations

The study was approved by the Institutional Review Board of Rajavithi Hospital (Approval No. 293/2021; approved on 17 December 2021). Verbal informed consent was obtained from all participants at the time of telephone interview, in accordance with the ethics committee approval.

Statistical analyses

Continuous variables are presented as mean ± SD or median and interquartile range (IQR) depending on distribution, while categorical variables are expressed as number and percentage (n (%)). Laboratory investigations were not available for all participants; therefore, percentages for laboratory variables were calculated based on available data. Comparisons between categorical variables were performed using the chi-square test or Fisher’s exact test, as appropriate. Continuous variables were compared using the Student’s t-test or Mann-Whitney U test based on data distribution. 

Statistical analyses were performed using Statistical Package for the Social Sciences (SPSS) software version 22.0. A p-value <0.05 was considered statistically significant. Given the relatively small number of outcome events, multivariable regression analysis was not performed because of concerns regarding model overfitting. Accordingly, the reported associations should be interpreted cautiously and considered exploratory.

## Results

Patient characteristics

A total of 324 patients with SLE were included. Most participants were women (94.4%), with a mean age of 40.8 ± 11.7 years. The median disease duration was 10 years (IQR 6-18). Baseline disease activity was low, with a median cSLEDAI-2K score of 0 (IQR 0-2); 65.7% were in clinical remission and 8.3% had LDA. Anti-dsDNA antibody and hypocomplementemia were present in 43.5% and 40.1% of patients, respectively.

Prednisolone was used by 288 patients (88.9%) at a median dose of 5 mg/day (IQR 2.5-7.5). Concomitant immunosuppressive therapy was prescribed in 218 patients (67.3%), including mycophenolate mofetil, cyclophosphamide, calcineurin inhibitors (tacrolimus or cyclosporine), azathioprine, and methotrexate. No patient received biologic therapy. The baseline demographic and clinical characteristics of the participants are summarized in Table 1.

**Table 1:** Baseline characteristics of SLE patients Values are presented as number (%), mean ± SD or median (IQR). ^† ^Include mycophenolate mofetil, azathioprine, methotrexate, cyclophosphamide, tacrolimus, and cyclosporine SLE: Systemic lupus erythematosus; cSLEDAI-2K: Clinical Systemic Lupus Erythematosus Disease Activity Index 2000; CR: Complete remission;  ESR: Erythrocyte sedimentation rate; eGFR: Estimated glomerular filtration rate; UPCR: Urine protein-creatinine ratio; aCL: Anti-cardiolipin antibody; IQR: Interquartile range

Characteristics	Total (n = 324)
Women	306 (94.4)
Age (years)	40.8 ± 11.7
Body mass index (kg/m^2^)	23.8 ± 5.4
Age at SLE diagnosis (years)	28.2 ± 12.1
Disease duration (years)	10.0 (6.0–18.0)
Comorbidities	
- Hypertension	45 (13.9)
- Dyslipidemia	19 (5.9)
- Chronic kidney disease	19 (5.9)
- Diabetes mellitus	9 (2.8)
- Cardiovascular disease	9 (2.8)
- Cerebrovascular disease	7 (2.2)
- Liver disease	5 (1.5)
cSLEDAI-2K	0 (0–2)
Disease activity	
CR	213 (65.7)
LDA	27 (8.3)
Not in CR/LDA	84 (25.9)
Antiphospholipid manifestations	26 (8.0)
Current medications	
Prednisolone	288 (88.9)
Hydroxychloroquine	240 (74.1)
Other immunosuppressive drugs^†^	218 (67.2)
Laboratory values	
ESR (mm/h)	27.8 (17.5–41.8)
Hemoglobin (g/dL)	12.1 ± 1.5
White cell count (x10^3^/μL)	6.8 ± 2.4
Platelet (x10^9^/L)	265 ± 77
Creatinine (mg/dL)	0.7 (0.6–0.9)
eGFR (mL/min/1.73 m^2^)	98.0 ± 31.7
UPCR (n = 233)	0.2 (0.1–0.2)
Elevated UPCR (> 0.5)	61 (26.2)
Anti-dsDNA antibody test (n = 62)	
Elevated anti-dsDNA (≥100 IU/mL)	27 (43.5)
Complement C3 test (n = 152)	
Low C3 (<0.83 g/L)	61 (40.1)
Antiphospholipid antibodies test (n = 44)	
Positive anti-β2GPI antibody	34 (77.2)
Positive aCL	18 (40.9)
Positive lupus anticoagulant	5 (11.4)
Other serologic tests (n = 62)	
Positive anti-Ro antibody	29 (46.8)
Positive anti-La antibody	10 (16.1)
Previous flare in the last 6 months	26 (8.0)
Immunosuppressive drug cessation before vaccination	31 (9.6)
Immunosuppressive drug cessation after vaccination	47 (14.5)

Patient-reported flares after vaccination 

Patient-reported flare symptoms occurred in 55 of 324 patients (17.0%). The median time to patient-reported flare onset after vaccination was seven days (IQR 2-14). The most common patient-reported flare manifestations were joint pain (41.8%), followed by hair loss (27.3%), fatigue (25.5%), myalgia, and fever (16.4%). The median joint pain severity score was 3 out of 10 (IQR 1-7). Some patients reported inflammatory features, including joint swelling and morning stiffness lasting up to 60 minutes. 34 patients (61.8%) reported having experienced similar flare symptoms in the past.

Treatment adjustment due to flare symptoms was required in 39 patients (70.9%), and hospitalization was required in five patients (9%). When analyzed by vaccine dose, treatment modification due to patient-reported flare occurred in 5.2%, 7.7%, and 7.0% of patients after the first, second, and third doses, respectively. Among the patient-reported flare group, nine (16.4%) experienced limitations in daily activities, and 12 (21.8%) required temporary work absence due to flare symptoms. Post-vaccination adverse events and patient-reported flare symptoms are presented in Table 2.

**Table 2:** Patient-reported SLE flare symptoms after COVID-19 vaccination Values are presented as number (%) or median (IQR). Percentages for flare manifestations were calculated based on the number of patients reporting flare symptoms after each vaccine dose (n = 19, 36, and 18 for the first, second, and third doses, respectively). The sum of per-dose values may exceed the total because a patient who experienced flares after more than one vaccine dose was counted in each respective dose column but only once in the total column. SLE: Systemic lupus erythematosus; IQR: Interquartile range

Variable	Total (324)	First vaccine (n = 324)	Second vaccine (n = 298)	Third vaccine (n = 214)
Patient-report flare	55 (17.0)	19 (5.9)	36 (12.0)	18 (8.4)
Joint pain	23 (41.8)	14 (73.7)	17 (47.2)	13 (72.2)
Hair loss	15 (27.3)	5 (26.3)	13 (36.1)	2 (11.1)
Fatigue	14 (25.5)	8 (42.1)	7 (19.4)	4 (22.2)
Myalgia	11 (20.0)	7 (36.8)	6 (16.7)	4 (22.2)
Fever	9 (16.4)	7 (36.8)	7 (19.4)	1 (5.6)
Dyspnea	9 (16.4)	6 (31.6)	7 (19.4)	1 (5.6)
Rash	7 (12.7)	2 (10.5)	6 (16.7)	2 (11.1)
Leg edema	7 (12.7)	4 (21.1)	6 (16.7)	1 (5.6)
Weakness	4 (7.3)	3 (15.8)	4 (11.1)	0 (0.0)
Oral ulcer	2 (3.6)	2 (10.5)	1 (2.8)	1 (5.6)
Seizure	1 (1.8)	1 (5.3)	0 (0.0)	1 (5.6)
Articular manifestations				
Tender joint count	2.0 (1.0–5.0)	2.5 (1.0–4.5)	2.0 (1.3–4.5)	1.0 (1.0–4.0)
Swollen joint count	1.0 (1.0–2.0)	1.0 (0–2.8)	1.5 (1.0–4.3)	1.0 (1.0–4.0)
Pain score	3.0 (1.0–7.0)	3.0 (1.0–8.0)	4.0 (1.0–8.0)	2.0 (1.0–6.3)
Time to flare onset after vaccination (days)	7.0 (2.0–14.0)	3.0 (1.0–7.0)	7.0 (2.0–14.0)	7.0 (1.0–14.0)
Previous similar flare symptoms	34 (61.8)	13 (68.4)	23 (63.9)	14 (77.8)
Drug adjustment	39 (70.9)	17 (89.5)	23 (63.9)	15 (83.3)
Hospitalization	5 (9.0)	4 (21.1)	2 (5.6)	2 (11.1)
Limit daily activities	9 (16.4)	4 (21.1)	7 (19.4)	4 (22.2)
Temporary work absence	12 (21.8)	5 (26.3)	8 (22.2)	4 (22.2)

Factors associated with patient-reported flare symptoms

Among the 55 patients in the patient-reported flare group, 54 (98.2%) were women, with a mean age of 38.9 ± 10.7 years. In the non-flare group (n = 269), 252 patients (93.7%) were women, with a mean age of 41.2 years. No significant differences were observed between the two groups in terms of age at diagnosis, disease duration, or baseline disease activity.

Current prednisolone use was associated with patient-reported flare symptoms (p = 0.049), though this borderline association should be interpreted cautiously given the exploratory nature of the analyses. No other clinical or treatment-related factors, including age, disease duration, baseline disease activity, or vaccine type, were associated with patient-reported flare (Table 3).

**Table 3:** Factors associated with patient-reported flare symptoms following COVID-19 vaccination Values are presented as number (%), mean ± SD or median (IQR). Laboratory data were not available for all participants. Percentages were calculated based on the number of patients with available data for each laboratory variable. † Include mycophenolate mofetil, azathioprine, methotrexate, cyclophosphamide, tacrolimus, and cyclosporine SLE: Systemic lupus erythematosus; cSLEDAI-2K: Clinical Systemic Lupus Erythematosus Disease Activity Index 2000; CR: Complete remission; LDA: Low disease activity; ESR: Erythrocyte sedimentation rate; eGFR: Estimated glomerular filtration rate; UPCR: Urine protein-creatinine ratio; aCL: Anti-cardiolipin antibody; IQR: Interquartile range

Characteristics	Flare (n = 55)	No flare (n = 269)	p-value
Female	54 (98.2)	252 (93.7)	0.222
Age (years)	38.9 ± 10.7	41.2 ± 11.9	0.195
Body mass index (kg/m^2^)	23.3 ± 5.9	23.9 ± 5.3	0.472
Age at SLE diagnosis (years)	25.8 ± 11.2	28.6 ± 12.2	0.105
Disease duration (years)	11 (7–18)	10 (5–18)	0.835
Comorbidities			
- Hypertension	8 (14.5)	37 (13.8)	0.853
- Dyslipidemia	5 (9.1)	14 (5.2)	0.337
- Chronic kidney disease	3 (5.5)	16 (5.9)	1.000
- Cerebrovascular disease	3 (5.5)	4 (1.5)	0.096
- Diabetes mellitus	2 (3.6)	7 (2.6)	0.651
- Cardiovascular disease	1 (1.8)	8 (3.0)	1.000
- Liver disease	1 (1.8)	4 (1.5)	1.000
cSLEDAI-2K	0 (0–0)	0 (0–0)	0.502
Disease activity status			0.630
CR	39 (70.9)	174 (64.7)	
LDA	3 (5.5)	24 (8.9)	
Not in CR/LDA	13 (23.6)	71 (26.4)	
Antiphospholipid manifestations	5 (9.4)	21 (7.7)	0.784
Current medications			
Prednisolone	53 (96.4)	237 (88.1)	0.049*
Hydroxychloroquine	40 (72.7)	200 (74.3)	0.902
Other immunosuppressive drugs^†^	41 (74.5)	177 (65.8)	0.122
Laboratory values			
ESR (mm/h)	26.5 (18.5–37.8)	28.5 (16.9–40.9)	0.112
Hemoglobin (g/dL)	12.2 ± 1.1	12.1 ± 1.5	0.662
WBC (x10^3^/μL)	6.8 ± 2.4	6.8 ± 2.4	0.889
WBC <4,000/mm^3^	6 (11.3)	19 (7.0)	0.228
Platelet (x10^9^/L)	266 ± 77	265 ± 77	0.979
Platelet <100,000/mm^3^	1 (1.9)	2 (0.7)	0.426
Creatinine (mg/dL)	0.7 (0.6–0.8)	0.7 (0.6–0.9)	0.294
eGFR (mL/min/1.73m^2^)	104.9 ± 27.6	96.7 ± 32.4	0.088
UPCR (n = 233)	0.3 (0.1–0.5)	0.2 (0.1–0.5)	0.592
Elevated UPCR (> 0.5)	9/37 (24.3)	52/196 (26.5)	0.779
Anti-dsDNA antibody test (n = 62)			
Elevated anti-dsDNA (≥100 IU/mL)	5/12 (41.7)	22/50 (44.0)	0.884
Complement C3 (n = 152)			
Low C3	13/26 (50.0)	48/126 (38.1)	0.260
Antiphospholipid antibodies			
Anti-β2GPI antibody test (n = 148)			
Positive anti-β2GPI antibody	6/23 (26.1)	28/125 (22.4)	0.778
aCL test (n = 156)			
Positive aCL	1/24 (4.2)	17/132 (12.9)	0.311
Lupus anticoagulant test (n = 22)			
Positive lupus anticoagulant	1/5 (20.0)	4/17 (23.5)	1.000
Other serologic tests			
Positive anti-Ro antibody (n = 62)	3/12 (25.0)	26/50 (52.0)	0.116
Positive Anti-La antibody (n = 62)	1/12 (8.3)	9/50 (18.0)	0.670
Previous flare in the past 6 months	5 (9.1)	21 (7.8)	0.785
Immunosuppressive drug discontinuation before vaccine	7 (12.7)	24 (8.9)	0.352
Immunosuppressive drug discontinuation after vaccine	11 (20.0)	36 (13.4)	0.214

We also analyzed the association between different COVID-19 vaccine types and the occurrence of patient-reported SLE flares. Most patients received at least two doses of COVID-19 vaccines (92.0%), and 66.0% received three doses. ChAdOx1-S was the most frequently administered vaccine for the first and second doses, followed by inactivated vaccines, according to the national vaccination program in the country. For the third dose, BNT162b2 was the most commonly administered vaccine. No significant association between vaccine type and patient-reported flare rate was observed (Table 4).

**Table 4:** Vaccine type and patient-reported SLE flare symptoms Data are presented as number (%). Percentages were calculated within flare and no-flare groups for each vaccine dose. SLE: Systemic lupus erythematosus

Vaccine	Flare	No flare	p-value
First vaccine (n = 324)	n = 19	n = 305	0.087
AstraZeneca	14 (73.7)	138 (45.2)	
Sinovac	3 (15.8)	72 (23.6)	
Sinopharm	2 (10.5)	57 (18.7)	
Pfizer-BioNTech	0 (0.0)	26 (8.5)	
Moderna	0 (0.0)	11 (3.6)	
Covovax (subunit)	0 (0.0)	1 (0.3)	
Second vaccine (n = 298)	n = 36	n = 262	0.118
AstraZeneca	21 (58.3)	142 (54.2)	
Pfizer-BioNTech	7 (19.4)	29 (11.1)	
Sinopharm	4 (11.1)	42 (16.0)	
Sinovac	3 (8.3)	39 (14.9)	
Moderna	1 (2.8)	10 (3.8)	
Third vaccine (n = 214)	n = 18	n = 196	0.531
AstraZeneca	2 (11.1)	28 (14.3)	
Pfizer-BioNTech	10 (55.6)	123 (62.8)	
Sinopharm	0 (0.0)	5 (2.6)	
Sinovac	0 (0.0)	1 (0.5)	
Moderna	6 (33.3)	39 (19.9)	

Adverse effects of COVID-19 vaccination

The most common vaccine-related adverse effect was injection-site pain, which occurred in 202 patients (62.3%). This was followed by fever, myalgia, fatigue, and headache, reported in 118 (36.4%), 43 (13.3%), 24 (7.4%), and 21 (6.5%) patients, respectively (Table 5). A total of 152 patients (46.9%) required medications to relieve adverse effects, including paracetamol, nonsteroidal anti-inflammatory drugs (NSAIDs), and antihistamines.

**Table 5:** Other adverse effects of COVID-19 vaccination Data are presented as number (%).

Side effect	Total (n = 324)
Total side effect	259 (79.9)
Injection-site pain	202 (62.3)
Fever	118 (36.4)
Myalgia	43 (13.3)
Fatigue	24 (7.4)
Headache	21 (6.5)
Nausea	12 (3.7)
Injection-site swelling	8 (2.4)
Diarrhea	6 (1.9)
Redness	3 (0.9)
Itching	2 (0.6)
Rash	2 (0.6)

One patient reported thrombosis following vaccination, and one patient reported pregnancy loss at a gestational age of 10 weeks.

## Discussion

Our findings contribute to real-world evidence regarding the safety profile of COVID-19 vaccination in patients with SLE. In this cross-sectional study of 324 patients with SLE, we observed patient-reported flare prevalence of 17.0% following COVID-19 vaccination. Our per-dose patient-reported flare rates (5.9-12.0%) were consistent with those observed in international cohorts (5.7-14.4%) [13,17,25,26].

Most patient-reported flares were not severe and predominantly musculoskeletal or constitutional, consistent with previous observations. Only 9% required hospitalization, without reported major organ manifestations, supporting the overall safety of repeated COVID-19 vaccination in SLE patients. The median onset of flare symptoms in our study was seven days post vaccination, which falls within the range reported in VACOLUP cohort and Kikuchi et al. [15,26].

Symptoms such as fatigue, myalgia, and arthralgia may overlap with common post-vaccination adverse effects. In the absence of physician confirmation or laboratory assessment, our findings should therefore be interpreted as patient-perceived disease worsening rather than confirmed immunologic flare. This distinction is important, as studies using validated flare indices, such as the VACOLUP cohort, have reported lower flare rates of 3% [15].

Current prednisolone use was the only factor associated with patient-reported flare symptoms, consistent with previous findings by González-Meléndez et al. [16]. However, this association may reflect underlying disease severity rather than a direct causal relationship with vaccination and should be interpreted cautiously because of the limited sample size, small number of patients not receiving prednisolone, and lack of multivariable adjustment. Other risk factors reported in previous studies [13,16] - such as high SLEDAI scores, and anti-dsDNA positivity - were not associated with patient-reported flare in our population, likely reflecting the high proportion of patients in clinical remission. No association was observed between patient-reported flares and vaccine type, consistent with findings from González-Meléndez et al. and Braverman et al. [14,16].

A recent physician-confirmed cohort from Northern Thailand by Louthrenoo et al. reported more severe flares, including renal involvement, using the Safety of Estrogens in Lupus Erythematosus National Assessment (SELENA) flare index [27]. While physician-confirmed studies capture organ-specific disease activity, our study provides complementary insight into patient-reported experiences and self-reported limitations in daily activities/work absence following vaccination.

Strengths of this study include the relatively large sample size, inclusion of multiple vaccine platforms (including inactivated vaccines, which are underrepresented in the literature), and evaluation across up to three vaccine doses. Additionally, the use of patient-reported outcomes reflects real-world experiences relevant to patient counseling. However, several limitations should be acknowledged. First, disease flares were self-reported and may have been overestimated due to symptom overlap with vaccine-related adverse effects. Second, the cross-sectional design with retrospective data collection may have introduced recall bias. Third, flare symptoms were assessed only within four weeks post-vaccination; delayed disease worsening occurring beyond this period may therefore not have been captured. Future studies incorporating longer follow-up periods are warranted to better characterize the full spectrum of post-vaccination disease course in SLE. Additionally, although different vaccine platforms may have distinct immunologic mechanisms and reactogenicity profiles, the number of patients receiving individual vaccine types was insufficient to permit meaningful vaccine-specific subgroup analyses. Findings regarding vaccine platform should therefore be interpreted with caution. Furthermore, multivariable regression analysis was not performed because of the limited number of outcome events; therefore, residual confounding may have influenced the observed associations, particularly the association between prednisolone use and patient-reported flare symptoms. Finally, laboratory confirmation of disease activity was not consistently available. Nevertheless, patient-reported experiences remain clinically relevant because perceived disease worsening may influence vaccine acceptance and adherence to booster recommendations in SLE. Nevertheless, this study provides real-world data regarding patient-reported experiences following COVID-19 vaccination in patients with SLE and contributes additional evidence regarding the tolerability of repeated vaccination in routine clinical practice.

## Conclusions

Patient-reported disease worsening following COVID-19 vaccination was relatively common in this cohort of patients with SLE; however, most reported symptoms were mild, with no reported organ-threatening manifestations. Joint pain, fatigue, and myalgia were the most frequently reported manifestations. Current prednisolone use was associated with patient-reported flare symptoms, although the clinical significance of this finding remains uncertain. No significant association was observed between vaccine platform and patient-reported flare symptoms, supporting the overall tolerability of repeated COVID-19 vaccination in patients with SLE.

These findings should be interpreted with caution because outcomes were based on patient-reported symptoms without standardized physician-confirmed flare assessment or systematic laboratory reassessment. In addition, the cross-sectional single-center design and exploratory statistical analyses may limit the generalizability of the findings. Nevertheless, this study provides real-world data regarding patient experiences after COVID-19 vaccination and may help support shared decision-making and vaccine confidence among patients with SLE. Further studies integrating both patient-reported outcomes and standardized clinical flare assessments may further improve understanding of the incidence, characteristics, and clinical relevance of post-vaccination disease worsening in this population.
